# Use of Rose Bengal, Methylene blue and Curcumin Photosensitizers activated using light emitting diode on post space disinfection bonded to fiber post: An assessment of extrusion bond strength

**DOI:** 10.12669/pjms.38.1.4780

**Published:** 2022

**Authors:** Fayez Hussain Niazi, Zeeshan Qamar, Mohammed Noushad, Abdullah Khaled Bin Muhareb

**Affiliations:** 1 Fayez Hussain Niazi, Department of Restorative and Prosthetic Dentistry, College of Dentistry, Dar Al Uloom University, Riyadh, Saudi Arabia; 2 Zeeshan Qamar, Department of OMFS and Diagnostic Sciences, Riyadh Elm University, Riyadh, Saudi Arabia. Department of Oral Biology, Liaquat College of Medicine Dentistry, Karachi. Pakistan; 3 Mohammed Noushad, Universiti Sains Malaysia, Kubang Kerian, Kelantan, Malaysia. Department of Restorative and Prosthetic Dentistry, College of Dentistry, Dar Al Uloom University, Riyadh, Saudi Arabia; 4 Abdullah Khaled Bin Muhareb, Ministry of Health, Kingdom of Saudi Arabia

**Keywords:** Canal disinfection, Curcumin, Extrusion bond strength, Methylene blue, Rose Bengal

## Abstract

**Objectives:**

To evaluate the effect of Rose Bengal (RB), methylene blue (MB) and curcumin used as a canal disinfectant on the extrusion bond strength (EBS) of FRCP (fiber reinforced composite resin) with canal dentin.

**Methods:**

The present invitro study was completed in 90 days approved by Riyadh Elm University. Forty premolars were extracted disinfected and decoronated. Mechanochemical preparation was done of canal space using 10k file widening canals sequentially with a 25K file with constant saline irrigation. Canal was dried condensed with gutta percha and sealer. Post space was prepared using peso reamer. Based on canal disinfection samples were divided into four groups. Group-1 MBP+17%EDTA, Group-2 RBP +17%EDTA, Group-3 Curcumin+17%EDTA and Group-4 5.25% NaOCl +17% EDTA. Following disinfection, the canal space of all specimens was washed with 17% EDTA for 120 sec. Post was cemented in canal space and cured. Specimens were placed on Universal testing machine (UTM) for EBS. The type of bond failure was evaluated using stereomicroscope. ANOVA and Tukey multiple comparison test was used to compare means.

**Results:**

Cervical third of Group-3 in which samples were disinfected with CP+17% EDTA displayed the maximum EBS (8.69±1.32 MPa). Whereas, the lowest EBS (3.30±0.54 MPa) was exhibited by the apical third of Group-4, where 5.25% NaOCl +17% EDTA was used as a canal disinfectant. The intragroup comparison demonstrated a declining trend of EBS from cervical to apical third in all investigated groups.

**Conclusion:**

Root canal dentin treated with different PS (MBP, CP, and RBP) demonstrated better EBS than the conventional disinfecting regime (NaOCl +17% EDTA). CP and RBP displayed better EBS than MBP

## INTRODUCTION

The prosthetic or restorative rehabilitation of root-treated teeth usually requires placement of post due to excessive compromise in clinical tooth structure.^1^ Among the different types of posts available, fiber-reinforced composite posts (FRCP) have gained acceptance due to favorable aesthetics, comparable modulus of elasticity to dentin, and acceptable biocompatibility.^2^ Moreover, the adhesive nature of FRCP contributes to the formation of a homogenous monoblock of dentin/post adhesive system which results in long-term clinical success Among various factors affecting FRCP in the canal space, the type and concentration of chemical disinfectant largely impact extrusion bond strength (EBS). Chemical disinfection along with mechanical debridement is considered to be a gold standard and widely used method for canal sterilization.^3^

Among the various chemical irrigants used in dentistry, sodium hypochlorite (NaOCl) had gained widespread popularity due to its antimicrobial effectiveness, tissue dissolving properties, and lubrication.^4^ It also possesses low viscosity which allows easy introduction and penetration into the canal space along with adequate shelf life and cost-effectiveness.^5^ However, existing literature advocates that NaOCl is unable to remove the inorganic component of the smear layer which barricades adhesion of bonded restorations.^6^ Moreover, it displays concentration-dependent cytotoxicity.^7^ Therefore, the use of chelator i.e., ethylenediaminetetraacetic acid (EDTA), and a mixture of doxycycline, acid, and detergent (MTAD) is being recommended as a final canal disinfectant after NaOCl to achieve complete smear layer removal. However, chemical irrigation contributes to bacterial load reduction yet does not disinfect the canal.^7^

Photodynamic therapy (PDT) has appeared to be a technical innovation that has modernized the concept of canal disinfection.^8^ PDT consist of light of a specific wavelength and photosensitizer (PS) which when absorbs by light generates free radical reactive oxygen species (ROS).^8^ In dentistry, multiple different photosensitizers i.e., methylene blue photosensitizer (MBP), tolonium chloride, photofrin, indocyanine green, and curcumin have been evaluated for their effect on shear bond strength (SBS), EBS, and microleakage. MBP also called methylthioninium chloride, is a cationic phenothiazine derivative.^9^ It becomes activated when irradiated with low-level laser therapy (LLT) with diode lasers at a wavelength of 660 nm. However, the literature showed contradictory outcomes related to its effect on EBS of fiber posts.^7^-^8^ CP on the other hand is a relatively new concept for canal disinfection obtained from turmeric powder and achieves antibacterial effectiveness similar to MBP by producing ROS.^9^ However, data related to its effect on PBS of FRCP is scarce. Recently, the role of xanthene-based photosensitizer Rose Bengal (RBP) has been investigated to evaluate its effects as an antimicrobial disinfectant against canal bacteria.^10^ It is an anionic red or rose color dye that belongs to the tetrachloro-tetraiodo derivative of fluorescein. It absorbs light in the wavelength range of visible light 500–800 nm.^11^ RB dye is considered an active PS as it generates a significant quantity of singlet oxygen species.

To our knowledge from existing indexed literature, it was found that the effect of NaOCl on the PBS of FRCP has been studied with conclusive outcomes. However, data related to the role of PDT on EBS of FRCP using different PS (MBP, CP, and RBP) is inconclusive with dubious outcomes. The existing study hypothesizes that PDT using different PS (MBP, CP, and RBP) will display comparable outcomes to PBS of FRCP when canal irrigated with conventional regimes. Therefore, the current in-vitro study aimed to evaluate the effect of different disinfecting regimes on the PBS of FRCP with canal dentin.

## METHODS

A sample of 40 extracted human premolars was collected from clinical settings. The present invitro study was completed in 90 days approved by Riyadh Elm University, FR-335m8. An ultrasonic scaler (Woodpecker U6 Scaler, USA) was used to remove the attached periodontal ligaments, inorganic and organic debris. All samples were stored in 0.4% thymol solution (Fórmula & Ação) maintaining the temperature at 4°C for 48 hours. Each specimen was de-coronated with a double-faced diamond disc (KG Sorensen, India) up to cementoenamel junction CEJ to standardized root length to 15 mm. The present study followed checklist for reporting invitro study CRIS guidelines.

All the samples were subjected to mechanochemical preparation of root canal by obtaining working length with a 10K size hand file (Dentsply; Maillefer Instruments, Ballaigues, Switzerland) keeping 1mm above the apical foramen. Canals were then widened by performing sequential filing up to size 25k file. This was followed by preparing canals with a rotary pro taper system till finishing file F3. During all the mechanical preparation root canals were repetitively irrigated by saline. The canal space of all specimens was dried using paper points (Dentex, Master Endo Dental Absorbent Paper Points) and made ready for obturation with gutta-percha (Radiant Surgident) and AH Plus sealer (Dentsply, Korea).

After root canal completion, peso-reamers (Dentmark, India) were used sequentially (2, 3, and 4) to prepare the post space. The gutta-percha was removed up to the length of 10 mm. All the post space were disinfected and were arbitrarily allocated in four different groups based on post space disinfecting agent used (n=10) each group

### Group-I:

MBP (Zhejiang Ronsheng Technology Co., Ltd) was used to disinfect the post space in this group. MBP was applied in the canal space for 180 sec before irradiation. MBP was activated using the fiber-optic tip of diode laser (Aarvam Medical Systems, India) at an incidence angle of 90° with 638nm wavelength, 30 Hz of frequency, and 2 watts of power.

### Group-II:

In this group samples were disinfected with 25 mol/L RB dye which is followed by irradiation with a green laser with peak absorptions at 540 nm. The activation time was of 60 sec. The method of activation was using the same fiber optic tip parallel to the long axis of the tooth.

### Group-III:

CP was placed as a post-space disinfectant in samples of this group. Photoactivation of CP was done using a light-emitting diode (LED) curing unit (Prototype, Finep/Gnatus LED Edixeon, Edison Opto Corporation, Taiwan) with the radiation intensity of 1200 mW/cm2.

### Group-IV:

In this group, irrigation with 5.25% NaOCl (Vardhman Chemi-Sol Industries, India) was performed using a 5ml 30-gauge needle for 60 sec in a to and fro motion.

Following disinfection, the canal space of all specimens was washed with 17% EDTA for 120 sec.

### Post Placement:

The disinfected post-space of all the specimens were dried with paper points. Within, the canal space glass fiber post (Swastik Dentomed Device, India) was placed after cleaning it with 70% ethanol. The post space was then air-dried and luted with the fiber post using Panavia F 2.0 (Kuraray Dental, Tokyo, Japan) self-etch dual-cure resin cement. The adhesive cement was cured by using LED (Elipar S10, 3M ESPE, Neuss, Germany). All the samples after cement polymerization were kept and stored for 24 hours in a dark environment maintaining the temperature at 37°C and humidity 100%

All the specimen were placed perpendicularly on resin blocks followed by sectioning at coronal, middle, and apical third (3mm each) using Isomet device (Isomet, Buehler, USA) under constant cooling. The sections were then placed in the universal testing machine (Shimadzu Corporation AutoGraph AGS-X Series, Kyoto-Japan) at 1mm/min crosshead speed. The highest force which was applied for extrusion of fiber post segment from the root dentin was measured in Mega Pascal (MPa). Modes of failure were identified with a Stereomicroscope at 40x magnification after EBS

### Statistical Analysis:

One-way analysis of variance (ANOVA) assessed the means and standard deviations (SD) of EBS. Tukey multiple comparison tests were used to compare means of EBS (*p*=0.05).

## RESULTS

Homogeneity of data was assessed using Kolmogorov-Smirnov test. Means and SD values among different experimental groups at the cervical, middle, and apical thirds of the root are displayed in Table-I. From the results, it was observed that the cervical third of Group-3 in which samples were disinfected with CP+17% EDTA displayed the maximum EBS (8.69±1.32 MPa). Whereas, the lowest EBS (3.30±0.54 MPa) was exhibited by the apical third of Group-4, where 5.25% NaOCl +17% EDTA was used as a canal disinfectant.

**Table I:** Details of photosensitizers used in the present study.

Photosensitizer	Charge	Excitation Maximum (nm)	The concentration of PS mg/L
Rose Bengal Photosensitizer	Anionic	559	100
Curcumin Photosensitizer	Neutral	547	500
Methylene Blue Photosensitizer	Cationic	632	100

Rose Bengal Photosensitizer (RB), Curcumin Photosensitizer (CP), Methylene Blue photosensitizer (MB).

**Fig.1:**
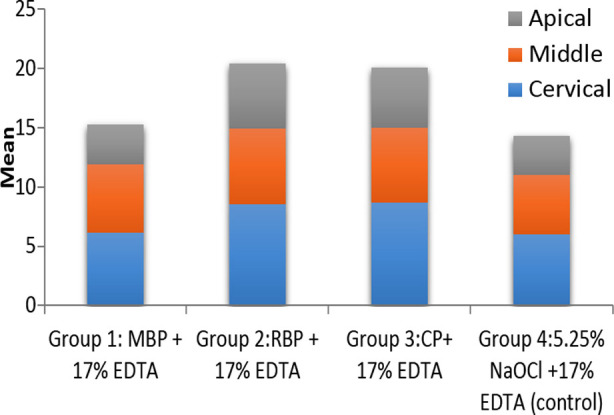
Means of PBS (MPa) values among experimental groups at the cervical, middle, and apical levels of roots. Sodium hypochlorite (NaOCl); Ethylene diamine tetraacetic acid (EDTA). Methylene blue photosensitizer (MBP), Rose Bengal photosensitizer(RBP), Curcumin photosensitizer (CP).

The intragroup comparison demonstrated a declining trend of EBS from cervical to apical third in all investigated groups. The EBS values of the cervical and middle third of all experimental groups were comparable (p>0.05). However, they were statistically different from apical thirds (*p*<0.05) (Table-I, Fig.1)

The intergroup comparison revealed comparable EBS of Group-2 specimens in which RBP + 17% EDTA was used as a canal disinfectant at all root levels (apical, middle, and coronal) with Group-3 (p>0.05). Whereas, Group-1 in which post space was irrigated with MBP + 17% EDTA and Group-4 demonstrated comparable extrusion bond values (p>0.05).

**Fig.2:**
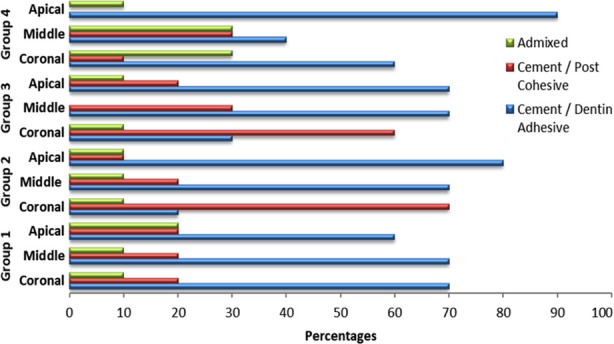
Pattern of fracture according to failure type.

Failure modes of all experimental groups are presented in Table-II. The majority of the failure mode was adhesive i.e., between cement and dentin. However, the middle third of Group-2 (RBP + 17% EDTA) and 3 (CP+ 17% EDTA) displayed a cohesive type of failure. (Fig.2)

**Table II:** Means and Standard deviations (SD) of Push-out bond strength (MPa) values among experimental groups at cervical, middle, and apical levels of root.

Groups	Cervical	Middle	Apical
Group-1: MBP + 17% EDTA	6.13±0.36 ^b, A^	5.75±0.22 ^b, A^	3.41±0.18 ^b, B^
Group-2: RBP + 17% EDTA	8.57±0.35 ^a, A^	6.38±1.11 ^a, A^	5.46±0.52 ^a, B^
Group-3: CP+ 17% EDTA	8.69±1.32 ^a, A^	6.30±0.83 ^a, A^	5.11±0.36 ^a, B^
Group-4: 5.25% NaOCl +17% EDTA (control)	6.01±1.37 ^b, A^	5.01±0.45 ^b, A^	3.30±0.54 ^b, B^

Sodium hypochlorite (NaOCl); Ethylene diamine tetraacetic acid (EDTA), Different superscript lower-case alphabets denote statistically significant difference within the same column (p<0.05), Data with different upper-case alphabets denotes significant difference within each row. (p<0.05).

## DISCUSSION

The present in vitro study was established on the hypothesis that PDT using different PS (MB, CP, and RB) will display comparable outcomes of FRCP EBS on radicular dentin irrigated with the conventional regime NaOCl+17% EDTA. To our surprise, the results of the current study revealed that specimens irrigated with PDT displayed better bond integrity of FRCP to root dentinal wall at all three levels (coronal, middle apical) than NaOCl+17% EDTA. Hence, the proposed hypothesis was rejected.

Bond integrity between the FRCP and a resin adhesive cement along with bond strength of resin luting agent with the radicular dentin wall is an important factor for clinical longevity and success of root restorative treatment.^12^-^13^ It was found from the results of the present study that 5.25% NaOCl +17% EDTA and MBP + 17% EDTA disinfected specimens showed the lowest and comparable EBS. This reduction in bond scores of self-etching adhesive to post space after irrigation with NaOCl +17% EDTA can be explained by alteration in the redox potential of root dentin due to NaOCl oxidization and the damage it causes to the organic component of dentin^7^-^14^ Hence, resin monomer would not be able to penetrate properly into the demineralized dentin which attributes to incomplete polymerization of resin luting cement thus compromising the bond integrity.^16^,^17^ This can be explained by the concentration of NaOCl used in the present study. Multiple previous studies have revealed that 5.25% of NaOCl decreases bond integrity of resin composite to caries affected dentin^6^,^17^,^18^, Similarly, a low EBS of MBP treated group can be justified because of the cationic character of the photosensitizer. These cationic molecules bind with anionic phosphate (P) and Calcium (Ca^+^) in dentinal structure resulting in modification of Ca^+^ and P ion ratio. This results in calcio-phosphate precipitation on the surface of canal dentin acting as a physical barrier for resin cement interaction with dentin.^13^ In addition, the hydrophilic nature of MBP also advocated low EBS achieved in the current study.^9.1.2.19^ Sayhon et al., in a study, explained that that absorbed water by MBP act as a barrier between resin and dentin thus compromising EBS values.^20^ The results of the existing study are following the work by Alonaizan et al.^21^

Furthermore, it was found that specimens, where CP and RBP were used as post space irrigants, displayed the highest and comparable EBS. A plausible explanation for these results would be the anionic nature of both the PS. However, CP inherently has neutral properties which upon dissociation forms anions of superoxide and hydrogen peroxide.^9^,.^19^ On the other hand, RBP is anionic by nature. Both the superoxide of Curcumin in dentin structure and RBP are attracted to cationic charged Ca^++^ ions thus causing surface alteration enhancing micromechanical retention of adhesive cement with improved bond integrity.^12^ Moreover, the hydrophobic nature of both CP and RBP along with hydrophobic cement possibly explains the outcome of better bond strength in these groups. Regarding modes of failure, both the MBP and NaOCl conditioned post space exhibited an adhesive type of failure when authenticates the lower bond integrity in these groups. Whereas, coronal sections of both CP and RBP disinfected samples displayed the cohesive failure which further confirms the higher EBS.^7^,^22^ However, the failure pattern was changed from cohesive to adhesive due to the difference in dentinal tubules from the cervical to the apical section of the root.^7^ The mode of failure observed in investigated groups harmonized with the EBS values.^23^.^24^

### Limitations of the study:

The current study exhibited some inherent methodological limitations. It is speculated that outcomes are influenced by the structural dentin variability at different locations i.e., tubular fluids, odontoblastic process along the root length. The presence of the smear layer, the difference in type and generation of the adhesive system used cannot be overlooked. Moreover, the effect of RBP on shear bond strength (SBS) and EBS either with FRCP with the root dentin or composite restorative material to the cavity dentin needs further investigation. Similarly, scanning electron microscopy (SEM) and Atomic force microscopy (AFM) of radicular dentin after CP and RBP need proper evaluation. It can also be understood that in vitro study design does not replicate the oral environment thus more in-vivo clinical trials are required to justify the finding of the present study.

## CONCLUSION

Root canal dentin treated with different PS (MBP, CP, and RBP) demonstrated better EBS than the conventional disinfecting regime (NaOCl +17% EDTA). CP and RBP displayed better EBS than MBP. Further, investigation is required in clinical settings to reciprocate the outcomes of the present study.

### Authors Contribution:

**FHA, ZQ:** Data collection, study design, manuscript writing, final manuscript approval.

**MN, ZQ:** Data collection, study design, manuscript drafting, data analysis, manuscript approval.

**FHA, MN:** Data collection, manuscript approval, and data interpretation.

**AKBM:** Data collection, writing, revise, editing, and final manuscript approval.

All authors are responsible and accountable for the accuracy and integrity of the work.
